# Patient Preferences in Neuromuscular Diseases: Insights for Future Drug Development

**DOI:** 10.1002/jmd2.70100

**Published:** 2026-06-01

**Authors:** Aura Cecilia Jimenez‐Moreno, Alasdair Blain, Cathy Anne Pinto, Vikas Soekhai, Jennifer Viberg Johansson, Christine Dyer, Kate Adcock, Esther W. de Bekker‐Grob, G. Ardine de Wit, Jane Newman, Gráinne S. Gorman

**Affiliations:** ^1^ Wellcome Centre for Mitochondrial Research. Translational and Clinical Research Institute. Faculty of Medical Sciences, Newcastle University Newcastle upon Tyne UK; ^2^ National Institute for Health and Care Research (NIHR) Newcastle Biomedical Research Centre (BRC) Newcastle upon Tyne UK; ^3^ Newcastle upon Tyne Hospitals NHS Foundation Trust Newcastle upon Tyne UK; ^4^ NHS Highly Specialised Service for Rare Mitochondrial Disorders of Adults and Children, Newcastle Upon Tyne Hospitals NHS Foundation Trust Newcastle upon Tyne UK; ^5^ Kielo Research York UK; ^6^ Merck & Co., Inc. Rahway New Jersey USA; ^7^ Erasmus Choice Modelling Centre, Erasmus University Rotterdam Rotterdam DR the Netherlands; ^8^ Erasmus School of Health Policy and Management, Erasmus University Rotterdam Rotterdam the Netherlands; ^9^ Department of Public Health and Caring Sciences Centre for Research Ethics and Bioethics, Uppsala University Uppsala Sweden; ^10^ Muscular Dystrophy UK London UK; ^11^ Julius Center for Health Sciences and Primary Care, University Medical Center Utrecht, Utrecht University Utrecht the Netherlands

**Keywords:** best worst scaling, mitochondrial myopathy, myotonic dystrophy, patient preferences, risk tolerance

## Abstract

Incorporating patient preferences into drug development is crucial, particularly, for rare diseases with significant unmet needs. This study used Best‐Worst Scaling type 2 (BWS‐2) to explore benefit–risk trade‐offs for patients and caregivers in two rare neuromuscular diseases (NMDs), myotonic dystrophy type 1 (DM1), and mitochondrial myopathy (MM). Patients with DM1 and MM, along with caregivers, completed a BWS‐2 survey assessing four treatment benefits (muscle strength, energy and endurance, balance, cognition) and two risks (permanent liver damage, temporary blurring of vision). Participants were stratified by disease group and age of onset (< 20, ≥ 20 years). A latent class analysis was used to calculate the relative importance of each treatment attribute. Sociodemographic and disease‐related data were also collected. A total of 270 participants (DM1 *n* = 143, MM *n* = 127, including 37 caregivers) were included. BWS‐2 results revealed a priority for improvements in muscle strength (24%), and energy and endurance (23%) across all groups, with caregivers placing a higher priority on cognition improvements (17%) compared to patients. There were no significant differences between disease groups or by age of onset. This study underscores the importance of patient preferences in drug development for rare NMDs. The consensus on treatment priorities across both diseases suggests that overlapping clinical features can inform and expedite future NMD or rare disease drug development.

## Take‐Home Message

This study highlights the importance of incorporating patient preferences into drug development for rare neuromuscular diseases. It reveals that patients and caregivers prioritize improvements in muscle strength, energy, and endurance over potential treatment risks.

**Abbreviations**
BWS‐2: Best‐Worst Scaling type 2
DCE: discrete choice experiment
DM1: myotonic dystrophy type 1
LCA: latent class analysis
MM: mitochondrial myopathies
NMD: neuromuscular disorders
PPI: patient preference information
RIS: Relative Importance Scores

## 1 Introduction

In recent years, measuring patient preferences has become an important scientific discipline [1]. Patient preference information (PPI) helps decision‐makers understand the unmet needs of patients, the potential value of new treatments, and the benefit–risk trade‐offs throughout the drug development [2]. Including patient perspectives can help overcome barriers in developing and regulating new drugs for rare diseases [3, 4].

Best‐Worst Scaling type 2 (BWS‐2) is an elicitation method for patient preferences in which respondents are asked to identify the best and worst options from a list (e.g., treatment characteristics) [5]. BWS‐2 allows for the precise quantification of preferences across multiple attributes, providing detailed insights into each attribute's relative importance [6]. This level of detail is crucial for understanding patients' and caregivers' specific needs and priorities.

Patient Preferences in Benefit–Risk Assessments during the Drug Life Cycle (PREFER) project aims to provide expert recommendations on how and when to use PPI in medical product decision‐making [7]. This project includes several case studies targeting different diseases and phases of drug development. Each case study combines qualitative and quantitative methods to understand patient preferences.

**FIGURE 1:**
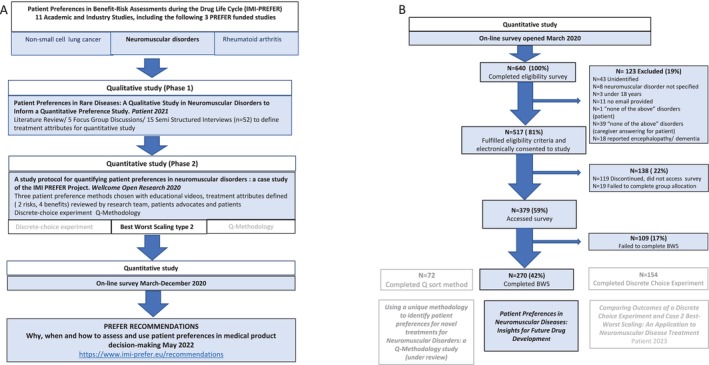
(A) Flow diagram outlining the phases of the IMI: PREFER NMD case study leading to the publication of prefer recommendations; (B) flow diagram of the quantitative part of the NMD case study. Details of the study reported here are represented in blue boxes. Details of the two other preference methods used in this case study are included in white boxes and grey font for understanding of the whole project.

In this study, we focused on two rare NMDs: myotonic dystrophy type 1 (DM1) and primary mitochondrial disease (MM). Both conditions have no curative treatments and may involve cognitive impairments [8, 9, 10]. We aimed to identify which disease features patients and caregivers find most burdensome and important for future drug development. We also quantified their preferences for a hypothetical medicine with both benefits and risks (Figure 1A). These insights could help guide and accelerate the development of new treatments for rare NMDs.

## 2 Methods

### 2.1 Protocol Approvals, and Patient Consents

The study protocol was approved by the Newcastle University R and D Ethics Committee (Ref: 15169/2018) and Newcastle Hospitals NHS Foundation Trust (Ref: 20/NW/0367; IRAS: 285949) [11]. Participants were recruited via patient registries in the United Kingdom, United States, Canada, Australia, New Zealand, and the Newcastle MitoCohort (MitoCohort application ref: MDOC ID065), with invitations sent including study information and data handling information. Eligibility and consent were confirmed online before the survey.

### 2.2 Study Participants

An international sample of patients with NMD and caregivers was invited to participate in an online survey. Participants were≥ 18 years old, with an active email account and self‐reported diagnosis of either DM1 or MM or were caregivers of such patients. Recruitment was supported by patient organizations and registries. Participants provided electronic informed consent and were stratified by age at diagnosis (< 20 years = Group 1, ≥ 20 years = Group 2) to differentiate those potentially more severely affected. Caregivers (spouse, partner, parent, legal guardian, or close relative) were asked to complete the survey based on the needs of the patient they cared for. Exclusions applied to those unable to consent, complete the survey, or with cognitive impairments (e.g., encephalopathy, dementia).

### 2.3 Survey Design and Measures

All participants completed the BWS‐2 (the focus of this manuscript). Basic demographic and clinical data were collected, alongside one other preference method. Activities of daily living were assessed using the ACTIVLIM‐Neuromuscular questionnaire, a patient‐reported questionnaire that evaluates performance in 22 daily tasks [12, 13]. Health literacy was measured using Chew's Brief Screening Questions and numeracy using the Subjective Numeracy Scale [14, 15].

The survey was pretested with eight cognitive interviews and refined based on pilot data from the first 50 participants. E‐learning modules were developed to explain the study and each preference method, using the example of a cough medicine, and pretested with patient representatives. Respondents only saw e‐learning relevant to their assigned stratum.

### 2.4 Patient Preference Elicitation Method

The survey aimed to assess patient treatment preferences using the BWS‐2 method [16, 17]. Unlike other methods, such as traditional rating scales or ranking methods, BWS‐2 forces respondents to prioritize and deprioritize attributes (i.e., treatment characteristics), leading to more discriminative and reliable data. This helps identify the most and least valued treatment attributes with greater accuracy. Given the diverse symptoms and severities within NMD populations, where it is not always realistic to provide treatments that can tackle all symptoms at once, BWS‐2 is well‐suited to capture a wide range of preferences, making it a robust method for studies involving heterogeneous patient groups. These advantages make BWS‐2 an ideal choice for this study, ensuring that the data collected is reliable and reflects true patient preferences.

The final BWS design included 16 choice tasks, which were divided into two blocks. Each block contained eight choice tasks and was randomly presented to respondents. The attribute order was kept consistent across all BWS‐2 tasks for each respondent to mitigate potential cognitive burden, but it was randomized across the sample [6]. Each choice task included six treatment attributes: four benefits (muscle strength, energy, balance, cognition) and two risks (temporary blurred vision, permanent liver damage) (Table 1).

**TABLE 1:** Descriptions of the six core treatment attributes (four benefits [muscle strength, energy, balance, cognition] and two risks [temporary blurred vision, permanent liver damage]) presented in the Best‐Worst Scaling type 2 survey for a hypothetical drug treatment.

Treatment attributes	Attribute description	Prompting question
		At this time in your life and given the specific treatment characteristics listed below, which characteristic do you consider the best option and which the worst of the treatment?
*Treatment benefits*	*These treatment options may improve the following …*	
Muscle strength	Disease‐related issues that may reduce your muscle strength interfering with your ability to lift things or to walk/run.	My muscle strength will be cured My muscle strength will be improved by half My muscle strength will stay the same
Energy and endurance	Disease‐related issues that may reduce your levels of energy and endurance when performing routine activities of the day (e.g., grocery shopping or a day at work or school) or when performing physical activity	My energy and endurance will be cured My energy and endurance will be improved by half My energy and endurance will stay the same
Balance	Disease‐related issues that may affect your balance, which may cause you to feel unsteady on your feet or lead to a fall.	My balance will be cured My balance will be improved by half My balance will stay the same
Cognition	Disease‐related issues that may interfere with your cognitive ability such as your capacity to remember things, concentrate, learn, or plan and perform new activities.	My cognition will be cured My cognition will be improved by half My cognition will stay the same
*Treatment risks*	*The treatment options may risk the following*	
Blurry vision	This side effect of treatment may cause your vision to become blurry. People with blurry vision are still able to walk but may not be allowed to drive or may have difficulty reading. However, if you stop taking the treatment the symptoms will gradually disappear.	99% chance of not experiencing **temporary** blurry vision 85% chance of not experiencing **temporary** blurry vision 70% chance of not experiencing **temporary** blurry vision
Liver damage	This side effect of treatment may cause your liver to stop functioning as normal. People with liver damage can experience fatigue and weakness. They can also experience yellowing of the skin and easy bruising. Unfortunately, this cannot be treated, and the damage will be permanent regardless of discontinuing the treatment.	99% chance of not experiencing **permanent** liver damage 85% chance of not experiencing **permanent** liver damage 70% chance of not experiencing **permanent** liver damage

*Note:* Participants were asked to choose their best and worst option from the combination of prompting questions. Benefits were presented in three possibilities using the prompting questions listed in the table, and the risks as a percentage of the chance of not experiencing the risk of the hypothetical treatment. Bold indicates groups are not compared for this variables.

**FIGURE 2:**
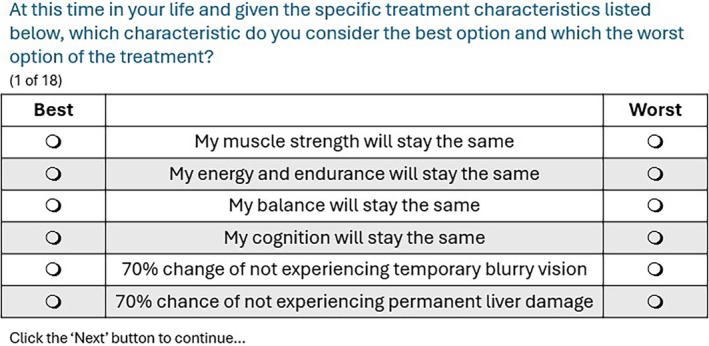
An example of BWS choice task as presented to participants in the survey.

The selection of attributes and levels was based on a previous qualitative study involving these two populations, gathering information on unmet health needs, reviewing clinical outcomes currently used in clinical trials within these groups, and finally validated through discussions with experts, including clinical key opinion leaders and patient representatives who are part of the research team. This previous qualitative study and the attributes prioritization process have been published earlier and are described in the study protocol [11, 18]. Benefits were presented in three levels (cured, improved by half, no change), and risks in three probabilities (70%, 85%, 99%) (Table 1), and participants were asked to choose the best and worst treatment attribute for them at each of the choice tasks [19] (Figure 2). Given prior qualitative findings indicating that participants may underestimate risks perceived as temporary, the severe side effect in this case (hepatic damage) was clearly described as permanent to ensure it was perceived as more severe.

### 2.5 Statistical Considerations and Data Analysis

Descriptive statistics summarized demographic and clinical data by disease type (DM1, MM) and age of onset (Group 1 < 20 years, Group 2 ≥ 20 years). Latent class analysis (LCA) was performed to identify subgroups with similar preferences [20]. Relative importance scores (RIS) were calculated using the range method, defined as the difference between the highest and lowest level coefficients within each attribute divided by the sum of these ranges across attributes.

Uncertainty in RIS was estimated using 10 000 parametric simulations of model coefficients assuming normal distributions defined by their estimated standard errors. Relative importance was recalculated for each simulation draw, and 95% confidence intervals were derived from the empirical 2.5th and 97.5th percentiles. As RIS is a nonlinear transformation of model coefficients, uncertainty was propagated using simulation rather than analytic standard errors.

The LCA model was implemented using the Apollo package in R (version 4.1.2).

## 3 Results

### 3.1 Clinical Demographics and Characteristics

Of 640 registered survey entries, 270 participants completed one full BWS‐2 survey and were therefore included in the analysis (Figure 1B). With a mean age of 51 years (range 18–85), most participants (78%, *n* = 210) were aged 25–64, and 35% (*n* = 95) were actively employed. Of the sample, 21% were unable to work due to disability. More than half (59%, *n* = 159) first reported symptoms after age 20 (Table S1). Over 95% of participants reported issues with muscle strength (*n* = 261) and energy and endurance (*n* = 259), while 85% (*n* = 240) experienced balance difficulties. Cognitive issues were reported by 13% (*n* = 34) of the total sample, but 87% (*n* = 32/37) of caregivers noted cognitive difficulties in patients. Liver problems and blurred vision were reported by 14% (*n* = 37) and 55% (*n* = 148), respectively (Table S2).

Disease severity varied: 26% (*n* = 69) could walk and run unaided, while 24% (*n* = 64) used a wheelchair (part‐ or full‐time). The average overall ACTIVLIM score across participants was 1.56 logits (Tables 2 and S3), with later onset participants (Group 2, mean 1.81 logits) showing less limitation in daily activities compared to earlier onset participants (Group 1, mean 0.95 logits). There were no significant differences in ACTIVLIM scores between DM1 and MM groups (DM1: mean 1.67, SD 2.58; MM: mean 1.44, SD 2.60). Over 25% of participants rated tasks such as “taking a bath” and “stepping out of a bath” as impossible. Caregivers reported significantly lower ACTIVLIM scores than patients (mean −0.40, SD 3.49, *p* < 0.001), with higher proportions reporting tasks like putting on a T‐shirt as impossible (22% caregivers vs. < 2% patients) (Table S3).

**TABLE 2:** Overall self‐reported ACTIVLIM scores—a logit is a linear unit that expresses the odds of success of the patient on any given item.

	Total number of participants	Onset of disease before 20 years old	Onset of disease after 20 years old	Caregiver group	Myotonic dystrophy (DM1)	Mitochondrial disease (MM)
Number of participants	270	69	159	37	143	127
Overall score (logits)						
Mean (SD)	1.56 (2.59)	0.95 (3.03)	1.81 (2.26)	−0.40 (3.49)	1.67 (2.58)	1.44 (2.60)
[Min; max]	[−6.56:5.07]	[−6.56:5.07]	[−4.18:5.07]	[−6.56:5.07]	[−6.56:5.07]	[−6.56:5.07]
Median [Q1; Q3]	1.56 [−0.22:3.45]	1.25 [−0.11:3.12]	1.56 [0.00:3.45]	−0.44 [−1.69:1.82]	1.56 [−0.22:3.45]	1.25 [−0.22:2.79]

*Note:* This scale is centered on the average item difficulty (0 logit). The less difficulty a patient experiences when executing activities of daily living, the more the measure will be positive in value. Bold indicates groups are not compared for this variables.

### 3.2 Health Literacy and Numeracy

Health literacy was similar between the two NMD groups, with an even split between high and low literacy levels. However, 84% of caregivers scored low in health literacy (Table S4). Numeracy scores were adequate across all groups, indicating average numeracy abilities (Table S5) [21].

### 3.3 Patient Preferences

**FIGURE 3:**
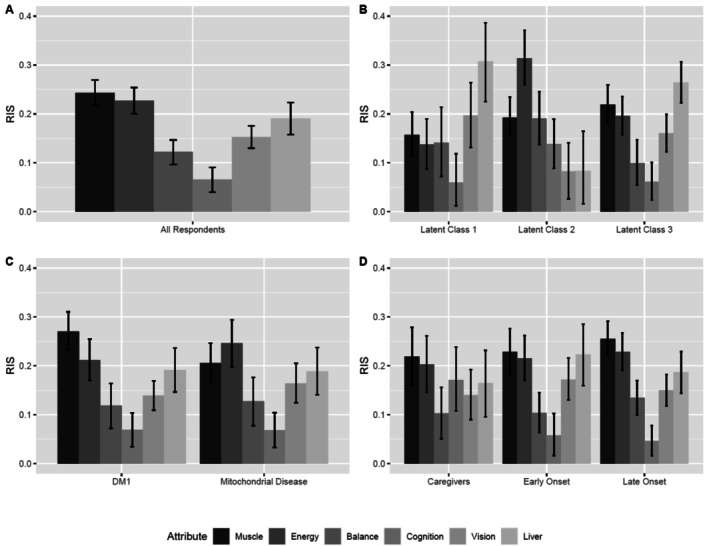
Graphical representation of Relative Importance Scores (RIS) for treatment attributes across respondent groups. Bars represent mean relative importance scores calculated using the range method. Error bars denote 95% confidence intervals derived from 10 000 parametric simulations of model coefficients. (A) All BWS respondents; (B) three latent classes; (C) DM1 and MM disease groups; (D) early and late onset groups and caregiver. The six attributes are shown in grey‐scale blocks in the key. A larger RIS score demonstrates a greater preference for this attribute to be treated by the hypothetical treatment.

Table 3 shows the relative weight of each attribute level from the BWS‐2, with higher benefits and lower risks preferred. Statistically significant differences (*p* < 0.01) were found for all attributes, with muscle strength and energy and endurance rated as the most important benefits. RIS for the six treatment attributes are shown in Figure 3A and Table 4.

**TABLE 3:** Coefficient estimates from a multinomial logit model of all participants (*n* = 270) completing the Best Worst Scaling Survey.

Risks and benefits of treatment	*N* = 270	
	*β* estimate	Robust standard error
Muscle strength		
Stays the same	Reference level	—
Improved by half	2.00***	0.17
Cured	2.99***	0.17
Energy and endurance		
Stays the same	Reference level	—
Improved by half	1.84***	0.17
Cured	2.78***	0.17
Balance		
Stays the same	Reference level	—
Improved by half	1.05***	0.11
Cured	1.71***	0.15
Cognition		
Stays the same	Reference level	—
Improved by half	0.83***	0.11
Cured	1.23***	0.14
Risk of blurry vision (%)		
1	Reference level	—
15	−0.45***	0.07
30	−0.80***	0.10
Risk of liver damage (%)		
1	Reference level	—
15	−1.15***	0.14
30	−1.51***	0.18
Reference levels		
Muscle strength: stays the same	0.00	—
Energy endurance: stays the same	−0.01	0.07
Balance: stays the same	0.21**	0.07
Cognition: stays the same	0.42***	0.09
Chance blurry vision (%): 1	1.08***	0.12
Chance liver damage (%): 1	0.83***	0.16

Abbreviation: *β* estimate = estimates attribute levels as additional utility or disutility compared to reference level.

* *p* < 0.05.

** *p* < 0.01.

*** *p* < 0.001.

**TABLE 4:** Relative importance scores (RIS) and 95% confidence intervals for treatment attributes across respondent groups.

Attribute—RIS (95% CI)	Overall	BWS1	BWS2	BWS3	DM1	MM	Onset 1	Onset 2	Care
Muscle	0.24 (0.22–0.27)	0.16 (0.11–0.20)	0.19 (0.15–0.23)	0.22 (0.18–0.26)	0.27 (0.23–0.31)	0.21 (0.17–0.25)	0.23 (0.18–0.28)	0.25 (0.22–0.29)	0.22 (0.16–0.28)
Energy	0.23 (0.20–0.25)	0.14 (0.09–0.19)	0.31 (0.26–0.37)	0.20 (0.16–0.24)	0.21 (0.17–0.25)	0.25 (0.20–0.29)	0.22 (0.17–0.26)	0.23 (0.19–0.27)	0.20 (0.15–0.26)
Balance	0.12 (0.10–0.15)	0.14 (0.07–0.21)	0.19 (0.14–0.25)	0.10 (0.05–0.15)	0.12 (0.07–0.16)	0.13 (0.08–0.18)	0.10 (0.06–0.15)	0.13 (0.10–0.17)	0.10 (0.05–0.16)
Cognition	0.07 (0.04–0.09)	0.06 (0.01–0.12)	0.14 (0.09–0.19)	0.06 (0.02–0.10)	0.07 (0.03–0.10)	0.07 (0.03–0.10)	0.06 (0.02–0.10)	0.05 (0.02–0.08)	0.17 (0.11–0.24)
Vision	0.15 (0.13–0.18)	0.20 (0.13–0.26)	0.08 (0.03–0.14)	0.16 (0.12–0.20)	0.14 (0.11–0.17)	0.16 (0.12–0.20)	0.17 (0.13–0.22)	0.15 (0.12–0.18)	0.14 (0.09–0.19)
Liver	0.19 (0.16–0.22)	0.31 (0.22–0.39)	0.08 (0.02–0.16)	0.26 (0.22–0.31)	0.19 (0.15–0.24)	0.19 (0.14–0.24)	0.22 (0.16–0.28)	0.19 (0.14–0.23)	0.17 (0.10–0.23)

Abbreviations: Care: caregivers' group; DM1: myotonic dystrophy type; Mito: mitochondrial disease; Onset 1: participants with early disease onset; Onset 2: participants with later disease onset.

### 3.4 LCA

LCA identified three distinct preference classes (Figure 3B). Class 1 prioritized avoiding liver damage and blurred vision; Class 2 favored energy and endurance improvements, and Class 3 showed a clear preference for muscle strength and energy and endurance benefits while avoiding the risks of blurred vision and liver damage. There were no significant differences in class membership by disease group (DM1 vs. MM, *p* = 0.1) or patient/caregiver status (*p* = 0.6), but a significantly higher proportion of participants with early onset disease (< 20 years) had a higher probability to belong to Class 3 (*p* = 0.03).

### 3.5 Comparison of Preferences by Disease and Group

Preferences between DM1 and MM were similar, with both groups placing high importance on muscle strength and energy and endurance (Figure 3C). Caregivers expressed a stronger preference for cognitive improvements compared to patients. Early onset patients (< 20 years) showed more divergent preferences, with larger coefficients for both their most and least preferred outcomes. Caregivers' preferences were generally aligned with later onset patients, except for a marked emphasis on cognitive improvement (Figure 3D).

## 4 Discussion

The PREFER NMD case study has been one of the first to quantitatively explore treatment preferences in patients with DM1 and MM using BWS‐2. This study adds valuable insight into patient preference research by examining the treatment priorities of individuals with these two rare neuromuscular diseases (NMDs), while also addressing the complexities introduced by cognitive impairment: an issue that complicates preference studies in these populations.

The study's primary objective was to quantify treatment preferences for hypothetical therapies aimed at improving key disease‐related symptoms. The results revealed that both DM1 and MM patients, as well as caregivers, highly valued improvements in muscle strength, energy, and endurance, with these attributes consistently ranked as the most important across all participant groups. This finding underscores the pressing unmet need for therapies targeting these symptoms, which have a significant impact on the daily lives of individuals with NMD. Moreover, while side effects were considered in the BWS‐2 method, participants were generally more concerned with the potential for improvement in these functional outcomes than with risks such as liver damage or blurred vision.

The heterogeneous nature of NMDs presents a challenge when designing preference studies, as patients with the same diagnosis can exhibit a wide range of symptoms [8, 9, 10]. Despite this, our study found that DM1 and MM patients shared many similar symptoms, including muscle weakness, fatigue, and poor balance, which allowed for a combined analysis. This finding suggests that phenotype (i.e., the observable symptoms and their severity) can be a more practical basis for grouping patients in preference studies than genotype alone. This approach could inform future research and trial design, helping to identify common treatment needs across conditions with overlapping clinical features. Our findings suggest that rare diseases, such as DM1 and MM, may benefit from a strategy of pooling patients with similar clinical features, regardless of genetic origin.

In 2017, the Myotonic Dystrophy Foundation submitted their findings from an externally led patient‐focused drug development work to the US Food and Drug Administration (FDA) [22]. In that report, they included results from a BWS with participants representing the myotonic dystrophy population (Types 1 and 2). Although the framing and attributes in that exercise differed from those included in this study, there is agreement on improving or maintaining muscle strength and avoiding the risk of liver failure.

In addition to patient preferences, the inclusion of caregivers in the study provided important insights into the impact of NMD on those who provide daily care. The caregiver group represented a more severe disease patients' profile, with lower scores on activities of daily living limitations and health literacy. Caregivers also reported a higher prevalence of cognitive difficulties in the patients they cared for, which may explain their stronger preference for treatments that could improve cognitive function. This highlights the essential role of caregivers in NMD research and emphasizes the importance of incorporating their perspectives into treatment development.

LCA identified three distinct preference patterns based on patients' preferences for treatment benefits and side effects. One preference pattern prioritized avoiding the risk of permanent liver damage, while another focused more on the potential benefits of energy and endurance improvements. A third preference pattern demonstrated a clear preference for improvements in muscle strength, and energy and endurance, with a strong aversion to side effects like blurred vision and liver damage. This finding suggests that patients vary in their risk tolerance and that treatment strategies should account for these differences. Tailoring treatment options based on patient preferences could increase acceptance and satisfaction with future therapies. Despite the significant contributions of this study, several limitations must be considered. First, the study did not reach the originally targeted sample size, which was based on the Discrete Choice Experiment (DCE) component. However, the BWS‐2, which was the focus of this study, allows a smaller sample size while still providing significant results, and the number of respondents was deemed sufficient for this analysis. Second, while the online survey allowed for a wide reach, it may have excluded individuals who were digitally disadvantaged or had physical impairments (e.g., visual impairment) that made online participation difficult. Additionally, the sample may not have fully captured the perspectives of the most severely affected patients, particularly those with cognitive impairment, as these patients may have been unable to complete the survey independently. The use of caregivers as proxies for these patients may have introduced bias, as caregivers may have struggled to separate their own preferences from those of the patients they cared for. Future studies should explore more inclusive recruitment strategies to reach underrepresented patient populations, including those with severe disease progression and pediatric patients.

Another potential limitation is the framing of side effects in the BWS‐2 method. Liver damage was framed as a permanent side effect, while blurred vision was described as temporary. LCA suggested that some patients placed significant importance on avoiding permanent side effects, which this framing may have influenced. The qualitative phase of the PREFER NMD study indicated that patients are particularly sensitive to the permanence of side effects, and this finding was reflected in the quantitative analysis [18]. This highlights the importance of how side effects are communicated to patients and suggests that the perceived permanence of a side effect may weigh more heavily in their decision‐making than the severity of the side effect itself.

## 5 Conclusion

The PREFER NMD case study provides important insights into the treatment preferences of patients with DM1 and MM. Key outcomes, such as muscle strength and energy and endurance, were consistently prioritized by both patients and caregivers. The study also highlights the feasibility of pooling patients with similar phenotypic expressions across different rare diseases, even when the underlying genetic etiology differs. This approach could inform future research and trial design, helping to identify common treatment needs across conditions with overlapping clinical features. The inclusion of caregivers also underscores the importance of considering their perspectives, especially for more severely affected patients. The findings from this study will be valuable for informing the design of future clinical trials and patient‐centered drug development strategies for rare diseases. By aligning treatment development with patients' benefit–risk trade‐offs, we can ensure that new therapies are better suited to meet the real‐world needs of those living with NMD. Additionally, the study contributes to the growing body of evidence on patient preferences in rare diseases and provides a framework for conducting similar research in other conditions with limited patient populations. Future work should focus on expanding the sample size, exploring additional patient subgroups, and refining recruitment strategies to ensure that all affected individuals are adequately represented.

## Funding

This study is part of the Patient Preferences in Benefit–Risk Assessments during the Drug Life Cycle (IMI‐PREFER) project. The PREFER project received funding from the Innovative Medicines Initiative 2 Joint Undertaking under grant agreement no. 115966. This Joint Undertaking receives support from the European Union's Horizon 2020 research and innovation program and the European Federation of Pharmaceutical Industries and Associations (EFPIA). The Wellcome Trust Award (203105/Z/16/Z) supports Professor Gorman's work.

## Conflicts of Interest

The authors declare no conflicts of interest.

## Supporting information


**Table S1:** Demographic data of all participants.
**Table S2:** Clinical characteristics in relation to disease attributes assessed with Best Worst Scaling‐2 questionnaire.
**Table S3:** Data reporting the difficultly of the individual tasks within the self‐reported ACTIVLIM questionnaire.
**Table S4:** Number and percentage of participants with high and low health literacy levels assessed using Chew's Set of Brief Screening Questions; low ≥ 3; high < 2. Please note a score of 2 is neither high nor low and hence not recorded in table.
**Table S5:** Health numeracy assessed using the Subjective Numeracy Scale (Score range 1–6 with higher scores reflecting higher numeracy).

## Data Availability

The data that support the findings of this study are available on request from the corresponding author. The data are not publicly available due to privacy or ethical restrictions.
