# Supplementary material for: Patient Preferences in Neuromuscular Diseases: Insights for Future Drug Development
Source: JIMD Rep. 2026 Jun 1;67(4):e70100. doi: 10.1002/jmd2.70100 (PMC13239944; doi:10.1002/jmd2.70100)
Supplement: Supplementary file 1 — Table S1: Demographic data of all participants. Table S2: Clinical characteristics in relation to disease attributes assessed with Best Worst Scaling‐2 questionnaire. Table S3: Data reporting the difficultly of the individual tasks within the self‐reported ACTIVLIM questionnaire. Table S4: Number and percentage of participants with high and low health literacy levels assessed using Chew's Set of Brief Screening Questions; low ≥ 3; high < 2. Please note a score of 2 is neither high nor low and hence not recorded in table. Table S5: Health numeracy assessed using the Subjective Numeracy Scale (Score range 1–6 with higher scores reflecting higher numeracy). [file JMD2-67-e70100-s001.docx]

Supplementary material

|  | **Total** | **Onset of disease before 20 years old** | **Onset of disease after 20 years old** | **Caregiver group** | **Myotonic Dystrophy (DM1)** | **Mitochondrial Disease**  **(MM)** |
| --- | --- | --- | --- | --- | --- | --- |
|  |  |  |  |  |  |  |
| **Number of participants** | **270** | **69** | **159** | **37** | **143** | **127** |
| **Age (in years)** |  |  |  |  |  |  |
| Mean (SD) | 51 (14) | 42 (16) | 54 (11) | 54 (13) | 49 (13) | 53 (15) |
| Range | 18:85 | 18:85 | 23:76 | 27:78 | 18:78 | 20:85 |
| **Age group** |  |  |  |  |  |  |
| 18 to 24 years (%) | 6 (2) | 4 (6) | 2 (1) | 0 (0) | 3 (2) | 3 (2) |
| 25 to 49 years | 114 (42) | 42 (61) | 54 (3) | 15 (41) | 69 (48) | 45 (35) |
| 50 to 64 years | 96 (36) | 15 (22) | 70 (44) | 11 (30) | 53 (37) | 44 (35) |
| 65 to 79 years | 52 (19) | 7 (10) | 33 (21) | 11 (30) | 18 (13) | 34 (27) |
| 80 or + years | 1 (<1) | 1 (1) | 0 (0) | 0 (0) | 0 (0) | 1 (1) |
| **Sex** |  |  |  |  |  |  |
| Male | 92 (34) | 21 (30) | 56 (35) | 14 (38) | 57 (41) | 35 (28) |
| Female | 178 (66) | 48 (70) | 103 (65) | 23 (62) | 82 (59) | 92 (72) |
| **Employment status** |  |  |  |  |  |  |
| Employed Full-time | 65 (24) | 20 (29) | 32 (20) | 11 (30) | 42 (29) | 23 (18) |
| Employed Part-time | 30 (11) | 7 (10) | 18 (11) | 4 (11) | 17 (12) | 13 (10) |
| Voluntary work | 13 (5) | 5 (7) | 7 (4) | 1 (3) | 4 (3) | 9 (7) |
| Unable to work or volunteer due to disability | 57 (21) | 23 (33) | 32 (20) | 2 (5) | 31 (22) | 26 (20) |
| Unemployed due to other reasons | 11 (4) | 2 (3) | 8 (5) | 1 (3) | 7 (5) | 4 (3) |
| Full-time student | 4 (1) | 1 (1) | 1 (1) | 1 (3) | 2 (1) | 2 (2) |
| Retired | 65 (24) | 7 (10) | 44 (28) | 13 (35) | 25 (17) | 40 (32) |
| Other | 24 (9) | 4 (6) | 16 (10) | 4 (11) | 14 (10) | 10 (8) |
| Prefer not to answer | 1 (<1) | 0 (0) | 1 (1) | 0 (0) | 1 (1) | 0 (0) |
| **Educational degree** |  |  |  |  |  |  |
| No mainstream formal schooling completed | 2 (1) | 1 (2) | 1 (0.63) | 0 (0) | 1 (<1) | 1 (0.8) |
| Attended a school for special education needs | 2 (1) | 1 (2) | 0 (0) | 1 (3) | 1 (<1) | 1 (0.8) |
| Attended a college for special educational needs | 1 (<1) | 0 (0) | 0 (0) | 1 (3) | 1 (<1) | 0 (0) |
| Attended formal schooling but left before age 14 | 0 (0) | 0 (0) | 0 (0) | 0 (0) | 0 (0) | 0 (0) |
| Attended formal schooling but left before age 14 | 12 (4) | 3 (5) | 8 (5) | 0 (0) | 5 (4) | 7 (6) |
| High school graduate, diploma or the equivalent | 47 (17) | 10 (18) | 28 (18) | 8 (22) | 28 (20) | 19 (15) |
| Some college credit or trade/technical/vocational training (A-levels in the UK) | 85 (31) | 7 (12) | 54 (34) | 11 (30) | 46 (32) | 39 (31) |
| Bachelor’s degree | 72 (27) | 21 (38) | 40 (25) | 9 (24) | 36 (25) | 36 (28) |
| Professional degree | 19 (7) | 2 (4) | 14 (9) | 3 (8) | 8 (6) | 11 (9) |
| Master’s degree | 22 (8) | 9 (16) | 11 (7) | 2 (5) | 12 (9) | 10 (8) |
| Doctorate degree | 8 (3) | 2 (4) | 3 (2) | 2 (5) | 5 (4) | 3 (2) |

S1 Table - Demographic data of all participants. Data is presented overall and split into various subgroups to allow comparison between the two different conditions, age of onset and with a caregivers group. The total is given for each option, with percentage of the total given in parentheses. **Where the total percentage does not sum to 100%, missing data has not been included**

|  | **Total** | **Onset of disease before 20 years old** | **Onset of disease after 20 years old** | **Caregiver group** | **Myotonic Dystrophy (DM1)** | **Mitochondrial Disease**  **(MM)** |
| --- | --- | --- | --- | --- | --- | --- |
|  |  |  |  |  |  |  |
| **Number of participants** | **270** | **69** | **159** | **37** | **143** | **125** |
| **Disease status** |  |  |  |  |  |  |
| Can walk AND run without the need of an assistive device (%) | 69 (26) | 22 (32) | 36 (23) | 8 (22) | 38 (27) | 31 (24) |
| Can walk without the need of an assistive device | 78 (29) | 14 (20) | 55 (35) | 7 (19) | 37 (26) | 41 (32) |
| Can walk but rely on an assistive device (i.e. walker, brace, cane, crutches, etc.) | 59 (22) | 12 (17) | 41 (26) | 6 (16) | 31 (22) | 28 (22) |
| Can walk (aided or unaided) but use a wheelchair part-time (e.g. long distances) | 45 (17) | 14 (20) | 23 (14) | 8 (22) | 29 (20) | 16 (13) |
| Rely fully on a wheelchair | 19 (7) | 7 (10) | 4 (3) | 8 (22) | 8 (6) | 11 (9) |
| **Muscle strength** |  |  |  |  |  |  |
| Not at all affected | 9 (3) | 1 (1) | 3 (2) | 2 (5) | 4 (3) | 5 (4) |
| Affected but only mildly | 127 (47) | 28 (41) | 83 (52) | 15 (41) | 67 (47) | 60 (47) |
| Affected severely | 134 (50) | 40 (58) | 73 (46) | 20 (54) | 72 (50) | 62 (49) |
| **Energy and endurance** |  |  |  |  |  |  |
| Not at all affected | 40 (15) | 1 (1) | 6 (4) | 2 (5) | 5 (3) | 6 (5) |
| Affected but only mildly | 121 (45) | 22 (32) | 75 (47) | 11 (30) | 67 (47) | 42 (33) |
| Affected severely | 109 (40) | 46 (67) | 78 (49) | 24 (65) | 71 (50) | 79 (62) |
| **Balance** |  |  |  |  |  |  |
| Not at all affected | 105 (39) | 8 (12) | 23 (14) | 7 (19) | 23 (16) | 17 (13) |
| Affected but only mildly | 131 (48) | 34 (49) | 74 (47) | 11 (30) | 58 (41) | 63 (50) |
| Affected severely | 34 (13) | 27 (39) | 62 (39) | 19 (51) | 62 (43) | 47 (37) |
| **Cognition** |  |  |  |  |  |  |
| Not at all affected | 105 (39) | 25 (36) | 72 (45) | 5 (13) | 61 (43) | 44 (35) |
| Affected but only mildly | 131 (48) | 35 (51) | 78 (49) | 17 (46) | 60 (42) | 71 (56) |
| Affected severely | 34 (13) | 9 (13) | 9 (6) | 15 (41) | 22 (15) | 12 (10) |
| **Liver damage** |  |  |  |  |  |  |
| Not aware of being affected | 232 (86) | 52 (75) | 140 (88) | 35 (95) | 127 (89) | 105 (83) |
| Liver affected but with no liver damage diagnosis | 24 (9) | 11 (16) | 11 (7) | 2 (5) | 11 (8) | 13 (10) |
| Have been diagnosed with liver damage | 13 (5) | 5 (7) | 8 (5) | 0 (0) | 4 (3) | 9 (7) |
| Is on the list for a liver transplant | 0 (0) | 0 (0) | 0 (0) | 0 (0) | 0 (0) | 0 (0) |
| Have had a liver transplant in the past | 1 (<1) | 1 (1) | 0 (0) | 0 (0) | 1 (<1) | 0 (0) |
| **Blurred Vision** |  |  |  |  |  |  |
| Not aware of being affected | 122 (45) | 31 (45) | 69 (43) | 18 (49) | 74 (52) | 48 (38) |
| Affected with impaired vision | 51 (19) | 16 (23) | 30 (19) | 5 (13) | 14 (10) | 37 (29) |
| Affected but treated or fixed using glasses | 97 (36) | 22 (32) | 60 (38) | 14 (38) | 55 (38) | 42 (34) |

*S2 Table - Clinical characteristics in relation to disease attributes assessed with Best Worst Scaling-2 questionnaire. The total is given for each option, with percentage of the total given in parentheses as whole numbers.* ***Where the total percentage does not sum to 100%, missing data has not been included.***

|  | **Total Number of participants** | **Onset of disease before 20 years old** | **Onset of disease after 20 years old** | **Caregiver** |  |  |
| --- | --- | --- | --- | --- | --- | --- |
|  |  |  |  |  | **DM1** | **MM** |
|  |  |  |  |  |  |  |
|  | **270** | **69** | **159** | **37** | **143** | **127** |
| **Putting on a T-shirt** | | | | | | |
| Impossible (%) | 10 (4) | 1 (1) | 1 (<1) | 8 (22) | 5 (3) | 5 (4) |
| Difficult | 71 (26) | 21 (30) | 35 (22) | 15 (40) | 41 (29) | 30 (24) |
| Easy | 189 (70) | 47 (68) | 123 (77) | 14 (38) | 97 (68) | 92 (72) |
| Unable to answer (?)* | 0 (0) | 0 (0) | 0 (0) | 0 (0) | 0 (0) | 0 (0) |
| **Washing one’s upper body** | | | | | | |
| Impossible | 11 (4) | 3 (4) | 0 (0) | 8 (22) | 6 (4) | 5 (4) |
| Difficult | 66 (24) | 21 (30) | 30 (19) | 15 (40) | 32 (22) | 34 (27) |
| Easy | 193 (72) | 45 (65) | 129 (81) | 14 (38) | 105 (73) | 88 (69) |
| Unable to answer (?)* | 0 (0) | 0 (0) | 0 (0) | 0 (0) | 0 (0) | 0 (0) |
| **Dressing one’s lower body** | | | | | | |
| Impossible | 20 (7) | 5 (7) | 4 (3) | 11 (30) | 12 (8) | 8 (6) |
| Difficult | 103 (38) | 30 (43) | 59 (37) | 14 (38) | 55 (39) | 48 (38) |
| Easy | 147 (54) | 34 (49) | 96 (60) | 12 (32) | 76 (53) | 71 (56) |
| Unable to answer (?)* | 0 (0) | 0 (0) | 0 (0) | 0 (0) | 0 (0) | 0 (0) |
| **Taking a shower** | | | | | | |
| Impossible | 26 (10) | 7 (10) | 6 (4) | 13 (35) | 13 (9) | 13 (10) |
| Difficult | 87 (32) | 25 (36) | 52 (33) | 10 (27) | 40 (28) | 47 (37) |
| Easy | 156 (58) | 36 (52) | 101 (63) | 14 (38) | 90 (63) | 66 (52) |
| Unable to answer (?)* | 1 (0.4) | 1 (1.4) | 0 (0) | 0 (0) | 0 (0) | 1 (0.8) |
| **Sitting on the toilet** | | | | | | |
| Impossible | 9 (3) | 3 (4) | 0 (0) | 6 (16) | 5 (3) | 4 (3) |
| Difficult | 50 (19) | 16 (23) | 21 (13) | 13 (35) | 28 (20) | 22 (17) |
| Easy | 211 (78) | 50 (73) | 138 (87) | 18 (49) | 110 (77) | 101 (80) |
| Unable to answer (?)* | 0 (0) | 0 (0) | 0 (0) | 0 (0) | 0 (0) | 0 (0) |
| **Taking a bath** | | | | | | |
| Impossible | 84 (31) | 21 (30) | 44 (28) | 19 (51) | 47 (33) | 37 (29) |
| Difficult | 57 (21) | 19 (27.5) | 34 (21) | 3 (8) | 22 (15) | 35 (28) |
| Easy | 114 (42) | 26 (38) | 71 (45) | 13 (35) | 66 (46) | 48 (38) |
| Unable to answer (?)* | 15 (6) | 3 (4) | 10 (6) | 2 (5) | 8 (6) | 7 (6) |
| **Walking downstairs** | | | | | | |
| Impossible | 51 (19) | 19 (28) | 20 (12) | 12 (32) | 26 (18) | 25 (20) |
| Difficult | 109 (40) | 21 (30) | 71 (45) | 16 (43) | 59 (41) | 50 (39) |
| Easy | 109 (40) | 28 (41) | 68 (4) | 9 (24) | 58 (41) | 51 (40) |
| Unable to answer (?)* | 1 (<1) | 1 (1) | 0 (0) | 0 (0) | 0 (0) | 1 (1) |
| **Stepping out of a bathtub** | | | | | | |
| Impossible | 83 (31) | 23 (33) | 42 (26) | 18 (49) | 42 (29) | 41 (32) |
| Difficult | 90 (33) | 23 (33) | 60 (38) | 7 (19) | 43 (30) | 47 (37) |
| Easy | 89 (33) | 22 (32) | 52 (33) | 10.(27) | 54 (38) | 35 (28) |
| Unable to answer (?)* | 8 (3) | 1 (1) | 5 (3) | 2 (5) | 4 (3) | 4 (3) |
| **Opening a door** | | | | | | |
| Impossible | 8 (3) | 1 (1) | 0 (0) | 7 (19) | 5 (3) | 3 (2) |
| Difficult | 51 (19) | 13 (19) | 28 (18) | 10.(27) | 30 (21) | 21 (17) |
| Easy | 211 (78) | 55 (80) | 131 (82) | 20 (54) | 108 (76) | 103 (81) |
| Unable to answer (?)* | 0 (0) | 0 (0) | 0 (0) | 0 (0) | 0 (0) | 0 (0) |
| **Walking outdoors on level ground** | | | | | | |
| Impossible | 25 (9) | 12 (17) | 4 (2) | 9 (24) | 9 (6) | 16 (13) |
| Difficult | 91 (34) | 22 (32) | 57 (36) | 12 (32) | 49 (34) | 42 (33) |
| Easy | 154 (57) | 35 (51) | 98 (62) | 16 (43) | 85 (59) | 69 (54) |
| Unable to answer (?)* | 0 (0) | 0 (0) | 0 (0) | 0 (0) | 0 (0) | 0 (0) |
| **Washing one’s face** | | | | | | |
| Impossible | 7 (3) | 2 (3) | 0 (0) | 5 (14) | 3 (2) | 4 (3) |
| Difficult | 39 (14) | 14 (20) | 12 (8) | 13 (35) | 18 (13) | 21 (17) |
| Easy | 224 (83) | 53 (77) | 147 (92) | 19 (51) | 122 (85) | 102 (80) |
| Unable to answer (?)* | 0 (0) | 0 (0) | 0 (0) | 0 (0) | 0 (0) | 0 (0) |
| **Hanging up a jacket on a hat stand or coat hook** | | | | | | |
| Impossible | 25 (9) | 8 (12) | 8 (5) | 9 (24) | 11 (8) | 14 (11) |
| Difficult | 57 (21) | 14 (20) | 30 (19) | 13 (35) | 35 (24) | 22 (17) |
| Easy | 188 (70) | 47 (68) | 121 (76) | 15 (41) | 97 (68) | 91 (72) |
| Unable to answer (?)* | 0 (0) | 0 (0) | 0 (0) | 0 (0) | 0 (0) | 0 (0) |
| **Wiping one's upper body** | | | | | | |
| Impossible | 12 (4.4) | 2 (2.9) | 3 (2) | 7 (19) | 6 (4) | 6 (5) |
| Difficult | 56 (20.7) | 18 (26.1) | 22 (14) | 16 (43) | 27 (19) | 29 (23) |
| Easy | 201 (74.4) | 49 (71.0) | 133 (84) | 14 (38) | 109 (76) | 92 (72) |
| Unable to answer (?)* | 1 (<1) | 0 (0) | 1 (<1) | 0 (0) | 1 (1) | 0 (0) |
| **Walking upstairs** | | | | | | |
| Impossible | 55 (20) | 18 (26) | 25 (16) | 12 (32) | 27 (19) | 28 (22) |
| Difficult | 139 (51) | 33 (48) | 87 (55) | 17 (46) | 74 (52) | 65 (51) |
| Easy | 75 (28) | 18 (26) | 46 (29) | 8 (22) | 41 (29) | 34 (27) |
| Unable to answer (?)* | 1 (<1) | 0 (0) | 1 (<1) | 0 (0) | 1 (<1) | 0 (0) |
| **Carrying a heavy load** | | | | | | |
| Impossible | 117 (43) | 36 (52) | 63 (40) | 18 (49) | 64 (45) | 53 (42) |
| Difficult | 112 (42) | 23 (33) | 75 (47) | 11 (30) | 57 (40) | 55 (43) |
| Easy | 29 (11) | 7 (10) | 19 (12) | 1 (3) | 15 (10) | 14 (11) |
| Unable to answer (?)* | 12 (4) | 3 (4) | 2 (1) | 7 (19) | 7 (5) | 5 (4) |
| **Getting into a car** | | | | | | |
| Impossible | 11 (4) | 3 (4) | 1 (1) | 7 (19) | 6 (4) | 5 (4) |
| Difficult | 106 (39) | 33 (48) | 62 (39) | 11 (30) | 53 (37) | 53 (42) |
| Easy | 142 (53) | 31 (45) | 94 (59) | 12 (32) | 77 (54) | 65 (51) |
| Unable to answer (?)* | 11 (4) | 2 (3) | 2 (1) | 7 (19) | 7 (5) | 4 (3) |
| **Standing for a long time (+ 10 min)** | | | | | | |
| Impossible | 69 (26) | 25 (36) | 32 (20) | 12 (32) | 37 (26) | 32 (25) |
| Difficult | 113 (42) | 26 (38) | 76 (48) | 10.(27) | 62 (43) | 51 (40) |
| Easy | 78 (29) | 16 (23) | 50 (31) | 8 (22) | 38 (27) | 40 (32) |
| Unable to answer (?)* | 10 (4) | 2 (3) | 1 (1) | 7 (19) | 6 (4) | 4 (3) |
| **Walking more than 1 kilometre** | | | | | | |
| Impossible | 94 (35) | 28 (41) | 51 (32) | 15 (41) | 52 (36) | 42 (33) |
| Difficult | 88 (33) | 19 (27) | 58 (36) | 10.(27) | 41 (29) | 47 (37) |
| Easy | 78 (29) | 20 (29) | 49 (31) | 5 (13) | 44 (31) | 34 (27) |
| Unable to answer (?)* | 10 (4) | 2 (3) | 1 (<1) | 7 (19) | 6 (4) | 4 (3) |
| **Closing a door** | | | | | | |
| Impossible | 2 (1) | 6 (9) | 0 (0) | 2 (5) | 1 (1) | 1 (1) |
| Difficult | 16 (6) | 16 (23) | 8 (5) | 2 (5) | 9 (6) | 7 (6) |
| Easy | 64 (24) | 47 (68) | 37 (23) | 8 (22) | 36 (25) | 28 (22) |
| Unable to answer (?)* | 188 (70) | 0 (0) | 114 (72) | 25 (68) | 97 (68) | 91 (72) |
| **Hopping on one foot** | | | | | | |
| Impossible | 42 (16) | 12 (17) | 23 (15) | 7 (19) | 23 (16) | 19 (15) |
| Difficult | 24 (9) | 4 (6) | 16 (10) | 3 (8) | 13 (9) | 11 (9) |
| Easy | 17 (6) | 6 (9) | 7 (4) | 2 (5) | 11 (8) | 6 (5) |
| Unable to answer (?)* | 187 (69) | 47 (68) | 113 (71) | 25 (68) | 96 (67) | 91 (72) |
| **Putting on a backpack** | | | | | | |
| Impossible | 14 (5.2) | 4 (5.8) | 6 (3.8) | 4 (10.8) | 8 (6) | 6 (5) |
| Difficult | 29 (10.7) | 8 (11.6) | 18 (11.1) | 3 (8.1) | 13 (9) | 16 (13) |
| Easy | 39 (14.4) | 10 (14.5) | 21 (13.2) | 5 (13.5) | 26 (18) | 13 (10) |
| Unable to answer (?)* | 188 (69.6) | 47 (68.1) | 114 (71.7) | 25 (67.6) | 96 (67) | 92 (72) |
| **Running** | | | | | | |
| Impossible | 43 (15.9) | 13 (18.8) | 24 (15.1) | 6 (16.2) | 22 (15) | 21 (17) |
| Difficult | 25 (9.3) | 4 (5.8) | 16 (10.1) | 4 (10.8) | 14 (10) | 11 (9) |
| Easy | 15 (5.6) | 5 (7.2) | 6 (3.8) | 2 (5.4) | 12 (8) | 3 (2) |
| Unable to answer (?)* | 187 (69.3) | 47 (68.1) | 113 (71.1) | 25 (67.6) | 95 (66) | 92 (72) |

*S3 table –Data reporting the difficultly of the individual tasks within the Self-reported ACTIVLIM questionnaire. The table reports how difficult the task was to perform from impossible to easy. Data is presented overall and split into various subgroups to allow comparison between the two different conditions, age of onset and with a caregivers group. The total is given for each question, with percentage of the total given in parentheses. * Unable to answer represented by a (?) indicated an activity that has not been attempted in the last three months by the participants and therefore they unable to estimate the difficulty.*

|  |  | **How often do you have someone help you read hospital materials?** | | **How confident are you filling out medical forms by yourself?** | | **How often do you have problems learning about your medical condition because of difficulty understanding written information?** | | **Overall** | |
| --- | --- | --- | --- | --- | --- | --- | --- | --- | --- |
|  |  |  |  |  |  |  |  |  |  |
|  |  |  |  |  |  |  |  |  |  |
| **Patient group** | **n** | **Low** | **High** | **Low** | **High** | **Low** | **High** | **Low** | **High** |
| All participants (%) | 270 | 79 (29) | 146 (54) | 85 (31) | 122 (45) | 74 (27) | 134 (50) | 123 (46) | 141 (52) |
| DM1 | 143 | 45 (31) | 76 (53) | 46 (32) | 65 (45) | 29 (20) | 84 (59) | 63 (44) | 76 (53) |
| MM | 127 | 34 (27) | 70 (56) | 39 (31) | 57 (45) | 45 (35) | 50 (40) | 60 (47) | 65 (51) |
| Early Onset of disease (<20yrs) | 69 | 22 (32) | 36 (52) | 24 (35) | 29 (42) | 25 (36) | 32 (46) | 36 (52) | 33 (48) |
| Later Onset of disease (>20yrs) | 159 | 30 (19) | 99 (62) | 38 (24) | 81 (51) | 29 (18) | 89 (56) | 56 (35) | 98 (62) |
| Caregivers | 37 | 27 (73) | 6 (16) | 23 (62) | 9 (24) | 20 (54) | 9 (24) | 31 (84) | 5 (14) |

S4 Table: Number and percentage of participants with high and low health literacy levels assessed using Chew’s Set of Brief Screening Questions; low ≥3; high <2. Please note a score of 2 is neither high nor low and hence not recorded in table.

| **Patient group** | **Average (Questions 1:8)** | **Ability (Questions 1:4)** | **Preference (Questions 5:8)** |
| --- | --- | --- | --- |
|  |  |  |  |
| All participants (n=270) | 3.793 | 3.894 | 3.692 |
| DM1 (n=143) | 3.728 | 3.809 | 3.647 |
| MM (n=127) | 3.794 | 3.742 | 3.846 |
| Early Onset of disease (<20yrs) (n=69) | 3.71 | 3.678 | 3.743 |
| Later Onset of disease (>20yrs) (n=159) | 3.951 | 4.016 | 3.887 |
| Caregivers (n=37) | 3.014 | 2.946 | 3.081 |

S5 Table: Health numeracy assessed using the Subjective Numeracy Scale (Score range 1-6 with higher scores reflecting higher numeracy)
